# Characteristics and outcome of severe traumatic brain injuries based on occupational status

**DOI:** 10.1007/s00068-020-01372-7

**Published:** 2020-04-18

**Authors:** Dominika Plancikova, Johannes Leitgeb, Alexandra Brazinova, Juliana Melichova, Patrik Sivco, Eva Nemcovska, Jarmila Pekarcikova, Marek Majdan

**Affiliations:** 1 Department of Public Health, Institute for Global Health and Epidemiology, Faculty of Health Sciences and Social Work, Trnava University, Hornopotocna 23, 91843 Trnava, Slovakia; 2 Department of Orthopedics and Trauma Surgery, Medical University of Vienna, Vienna, Austria; 3 International Neurotrauma Research Organisation, Vienna, Austria; 4 Faculty of Medicine, Comenius University, Bratislava, Slovakia

**Keywords:** Traumatic brain injury, Outcome, Occupational status, Epidemiology

## Abstract

**Purpose:**

The association of TBI with socioeconomic characteristics of patients has not been studied extensively. The objective of this study was to analyse the differences in injury characteristics and outcome in TBI patients based on their occupational status.

**Methods:**

Data on patients from 13 centres based in Austria, Croatia, Slovakia, Bosnia and Herzegovina, and Macedonia were included in the analysis. Demographic characteristics, injury characteristics, treatment and outcome at various post-injury stages were compared according to occupational status. Logistic regression was used to adjust for the effect of co-variates. ICU mortality, hospital mortality, 6 months mortality, and outcome at 6 months were used as dependent variables.

**Results:**

Overall, 886 patients were analysed with a mean age of 45.5 years. High-level falls were most prevalent in the blue-collar group (19%), most low-level falls occurred in the retired group. Traffic accidents were most common in students. The injuries were most severe in the blue-collar group and students. Highest mortalities and unfavourable outcomes were in the retired, students and white-collar workers had the best outcomes. Compared to retired patients, all groups had higher odds of favourable outcome at 6 months after adjusting for co-variates—OR from 2.2 (95% CI 1.1–4.6) for entrepreneurs to 3.6 (95% CI 1.8–7.2) for the blue-collar group.

**Conclusion:**

Our paper provides clues pertaining specifically to variations in patterns and outcomes of TBI according to occupational status which can inform prevention and planning of services and can serve to plan priorities for further research.

## Purpose

Traumatic brain injuries (TBI) have been identified as a major public health problem [1, 2]. They are among the most important causes of morbidity and mortality among all age groups globally, they pose substantial burden on victims, their families and society as a whole [3]. In Europe alone, a recent analysis estimated the hospital-based incidence of TBI at 284 and the mortality at 11 per 100,000 population, which translates to about 1.5 million hospital admissions and 57,000 deaths [1]; an average TBI-related death in Europe was associated with about 24 years of lost life [4]. The global burden of diseases study estimated the global incidence of TBI at 369 per 100,000 which means that an estimated 27.08 million new TBI cases occurred in 2016 [2].

Socioeconomic status (SES) is in general considered to be an important determinant of general morbidity and mortality [5–7]. However, research on the association of SES with outcomes after injuries received only little attention until recent years [8], with studies focusing on TBI being even less common and relatively restricted as to study population, studied outcomes, or severity groups [9–13].

Most published studies of associations between SES and injuries focus on paediatric populations with fewer using cohorts of TBI cases from general populations, and their results are conflicting: some studies showed a longer stay in hospital [14], increased 30 day mortality [15], higher risk of injury death [16] and worse non-fatal outcome [8] in more deprived patients; some studies showed associations only in certain subtypes of injuries, such as fighting injuries or sports/recreational injuries [17]; others did not observe any significant differences in outcome based on SES [13, 18, 19].

Previous studies have shown that the context of the injury such as mechanism [20], place of occurrence of the injury [21], age group or social group [22] shows distinctive patterns as to severity, extent and overall outcome of TBI. In a similar manner we hypothesized that TBI will display distinctive patterns of cause, occurrence and outcome based on SES. For our study we have used occupational status as a proxy for SES, as this has been shown to be well approximating SES in general [7].

The objective of this study was to analyse the differences in injury characteristics, treatment, and outcome at various stages post-injury in 886 patients with TBI based on the occupational status of the patient.

## Methods

### Data collection and characteristics

Data on patients from 13 centres were included in the analysis. These data were collected between January 2001 and June 2005 from centres based in Austria (Graz, Klagenfurt, Linz, Salzburg, Vienna), Bosnia and Herzegovina (Sarajevo), Croatia (Osijek, Rijeka, Zagreb), Macedonia (Skopje) and Slovakia (Banska Bystrica, Martin, Michalovce). Data were collected by using the International Traumatic Coma Project [23] database. Data on a total of 1104 patients were available. Of these, data on social status and occupation were available in 886 patients and these were used in this study.

Only patients who sustained severe TBI and survived at least until admission to the intensive care unit (ICU) were included in the dataset—severe TBI was defined as a Glasgow Coma Scale (GCS) score of 8 or less following resuscitation or a GCS score deteriorating to 8 or less within 48 h of injury.

Detailed data on demographic characteristics (age, sex, education), injury characteristics [mechanism of injury, abbreviated injury scale (AIS) for the region of head, first GCS, Injury Severity Score (ISS), main diagnosis], treatment factors [such as mode of transport, intubation, intracranial pressure (ICP) monitoring, surgeries, days at ICU, length of hospital stay], and outcome at various stages post-injury (ICU mortality, hospital mortality, 6 months mortality, and GOS at 6 months) were available and used in the analyses within this study.

Six-month outcome was recoded from GOS to favourable outcome (GOS of 5 or 4) or unfavourable outcome (GOS of 3 or less). Main diagnosis was stated as contusion, oedema, epidural haemorrhage (EDH), intraventricular haemorrhage (IVH), normal, subarachnoid haemorrhage (SAH), subdural hematoma (SDH) or unknown.

### Analysis outline

The main line of analysis within this study was to compare demographic characteristics, injury characteristics, treatment modalities and outcome at various stages post-injury using occupational status at the time of injury as a stratification variable. For this purpose, six occupational categories were created: blue-collar workers, white-collar workers, entrepreneurs, retired, unemployed and students. First, all available factors were compared across the six groups using univariate analyses—Chi-squared test or Kruskal–Wallis test was used as appropriate.

Subsequently, multivariable logistic regression models were constructed to adjust the association between occupational status and outcomes for the effect of co-variates. Mortality at ICU discharge, mortality at hospital discharge, 6 months mortality and outcome at 6 months post-injury (favourable/unfavourable) were used as dependent variables. Nagelkerke’s *R*^2^ and AUC were calculated to determine the characteristics of the models. *P* value < 0.05 was assumed statistically significant. All statistical analyses were performed in R-software [24].

## Results

Demographic characteristics of the patients are presented in Table 1. Overall, 886 patients were included in this analysis. Patients were distributed into the groups quite evenly, retired being the largest (213, 24%) and entrepreneurs the smallest (93, 10%). The overall mean age was 45.5 years (SD = 21.3), entrepreneurs being the oldest of the three economically active groups (i.e. blue- and white-collars, and entrepreneurs). Male sex dominated each group. University-level education was most prevalent in the white-collar and entrepreneur group, almost half of the blue-collar and unemployed group only reached elementary level education. Overall, the distribution of age, sex and education was significantly different among the compared groups.

**Table 1:** Demographic characteristics of TBI patients by occupational status

Variable	Occupational status						Total (*n* = 886)	*P* value
	Blue-collar (*n *= 168)	White-collar (n = 105)	Entrepreneur (*n* = 93)	Retired (*n* = 213)	Unemployed (*n* = 134)	Student (*n* = 173)		
Age (mean, SD)	40.2 (12.1)	37.9 (13.3)	45 (10.8)	70.5 (10.3)	53.5 (17.5)	18.4 (4.8)	45.5 (21.3)	< 0.001
Sex (*n*, % male)	155 (92%)	80 (76%)	80 (86%)	157 (74%)	81 (60%)	134 (78%)	687 (78%)	< 0.001
Education (*n*, %)								
University	4 (2%)	19 (18%)	13 (14%)	15 (7%)	5 (4%)	25 (14%)	81 (9%)	< 0.001
High school	45 (27%)	24 (23%)	38 (41%)	33 (16%)	23 (17%)	69 (40%)	232 (26%)	
Vocational	32 (19%)	58 (54%)	24 (25%)	23 (11%)	7 (5%)	5 (3%)	149 (17%)	
Elementary	69 (41%)	1 (1%)	14 (15%)	35 (16%)	56 (42%)	66 (38%)	241 (27%)	
Unknown	18 (11%)	3 (3%)	4 (4%)	107 (50%)	43 (32%)	8 (5%)	183 (21%)	

*SD* standard deviation

Data on injury characteristics are presented in Table 2. Injury mechanism distributions were significantly different among the six groups. Low-level falls (231, 26%), traffic accidents of drivers (121, 14%) and of pedestrians (100, 11%) were the most common in general. High-level falls were most prevalent in the blue-collar group (32, 19%), whereas, most low-level falls occurred in the retired group. Retired and unemployed had the lowest proportion of traffic-related accidents. Traffic-related accidents (all road users combined) were most common in the student group.

**Table 2:** Injury mechanism and injury severity characteristics of TBI patients by occupational status

Variable	Occupational status						Total (*n* = 886)	*P* value
	Blue-collar (*n* = 168)	White-collar (*n* = 105)	Entrepreneur (*n* = 93)	Retired (*n *= 213)	Unemployed (*n* = 134)	Student (*n* = 173)		
Mechanism (*n*, %)								< 0.001
Fall < 3 m	31 (18%)	15 (14%)	15 (16%)	97 (46%)	60 (45%)	13 (8%)	231 (26%)	
Fall > 3 m	32 (19%)	6 (6%)	9 (10%)	11 (5%)	10 (7%)	9 (5%)	77 (9%)	
Traffic (driver)	27 (16%)	31 (30%)	22 (24%)	4 (2%)	10 (7%)	27 (16%)	121 (14%)	
Traffic (passenger)	6 (4%)	5 (5%)	10 (11%)	11 (5%)	7 (5%)	31 (18%)	70 (8%)	
Traffic (pedestrian)	10 (6%)	8 (8%)	4 (4%)	31 (15%)	15 (11%)	32 (18%)	100 (11%)	
Traffic (bicycle)	5 (3%)	6 (6%)	0	13 (6%)	3 (2%)	9 (5%)	36 (4%)	
Traffic (motorcycle)	19 (11%)	9 (9%)	4 (4%)	6 (3%)	3 (2%)	31 (18%)	72 (8%)	
Assault	9 (5%)	4 (4%)	7 (8%)	4 (2%)	5 (4%)	4 (2%)	33 (4%)	
Gunshot	7 (4%)	5 (5%)	5 (5%)	9 (4%)	5 (4%)	8 (5%)	39 (4%)	
Other	14 (8%)	12 (11%)	10 (11%)	15 (7%)	6 (4%)	4 (2%)	61 (7%)	
Unknown	8 (5%)	4 (4%)	7 (8%)	12 (6%)	10 (7%)	5 (3%)	46 (5%)	
AIS head (mean, SD)	4.2 (0.9)	4.1 (1)	3.5 (1.2)	4.2 (1)	4.1 (1.2)	3.9 (1.1)	4 (1.1)	< 0.001
First GCS (mean, SD)	6 (2.7)	5.8 (2.9)	6.7 (3.3)	5.9 (2.9)	5.9 (2.8)	5.9 (2.9)	6 (2.9)	0.238
ISS (mean, SD)	32.4 (16.2)	29.7 (15.7)	25.2 (14)	26.8 (15)	29.4 (18.8)	33.9 (15.9)	29.8 (16.2)	< 0.001
Main diagnosis (*n*, %)								< 0.001
Contusion	27 (16%)	22 (21%)	18 (19%)	24 (11%)	13 (10%)	28 (16%)	132 (15%)	
Oedema	3 (2%)	6 (6%)	1 (1%)	1 (0%)	2 (1%)	6 (3%)	19 (2%)	
EDH	34 (20%)	20 (19%)	6 (6%)	12 (6%)	21 (16%)	31 (18%)	124 (14%)	
IVH	23 (14%)	11 (10%)	18 (19%)	32 (15%)	14 (10%)	49 (28%)	147 (17%)	
Normal	6 (4%)	4 (4%)	2 (2%)	3 (1%)	1 (1%)	6 (3%)	22 (2%)	
SAH	8 (5%)	6 (6%)	4 (4%)	10 (5%)	6 (4%)	6 (3%)	40 (5%)	
SDH	62 (37%)	34 (32%)	43 (46%)	129 (61%)	75 (56%)	44 (25%)	387 (44%)	
Unknown	5 (3%)	2 (2%)	1 (1%)	2 (1%)	2 (1%)	3 (2%)	15 (2%)	

*AIS* Abbreviated Injury Scale, *SD* standard deviation, *GCS* Glasgow Coma Scale, *ISS* Injury Severity Score, *EDH* epidural hematoma, *IVH* intraventricular haemorrhage, *SAH* subarachnoid haemorrhage, *SDH* subdural hematoma

Although the GCS did not differ significantly among the groups, the ISS suggest that the overall injuries were significantly more severe in the blue-collar group, and the student group—compared to all other. The head AIS of the head suggests that head injuries in the entrepreneur and student groups were somewhat less severe, compared to the rest of the groups. SDH were the most common diagnosis across the groups, followed by IVH, contusions, and EDH.

Table 3 presents a summary of treatment modalities. Air transport was significantly more common in the blue- and white-collar groups, compared to the rest. ICP monitoring was least common in the entrepreneur group (23, 25%), cranial surgeries had to be performed most commonly in white-collar and unemployed group (72, 69% and 96, 72%, respectively). White-collar workers had significantly the highest median stay at ICU and at hospital.

**Table 3:** Treatment factors of TBI patients by occupational status

Variable	Occupational status						Total (*n* = 886)	*P* value
	Blue-collar (*n* = 168)	White-collar (*n* = 105)	Entrepreneur (*n* = 93)	Retired (*n* = 213)	Unemployed (*n* = 134)	Student (*n *= 173)		
Air transport (*N*, % Yes)	34 (20%)	32 (30%)	11 (12%)	26 (12%)	14 (10%)	11 (6%)	128 (14%)	< 0.001
Intubation (*N*, % Yes)	100 (60%)	80 (76%)	53 (57%)	115 (54%)	67 (50%)	82 (47%)	497 (56%)	< 0.001
ICP monitoring (*N*, % Yes)	82 (49%)	63 (60%)	23 (25%)	91 (43%)	64 (48%)	61 (35%)	384 (43%)	< 0.001
Days ICP monitoring (mean, SD)	8 (5.9)	8.8 (5.3)	6.2 (3.3)	7 (5.5)	6.2 (4.6)	7.8 (5.2)	7.4 (5.3)	< 0.058
Cranial surgery (*N*, % yes)	101 (60%)	72 (69%)	50 (54%)	135 (63%)	96 (72%)	80 (46%)	534 (60%)	< 0.01
Days at ICU (median, IQR)	9 (4–19)	11 (5–26)	5 (2–14)	7 (3–13)	7 (3–12)	7 (3–11)	8 (3–15)	< 0.001
Hospital stay (median, IQR)	17 (6–30)	19 (6–41)	10 (3–28)	8 (4–20)	11 (3–33)	14 (5–25)	12 (4–28)	< 0.001

*ICP* intracranial pressure, *IQR* interquartile range, *ICU* intensive care unit

The outcome patterns are presented in Table 4. In general, the mortalities at any point in time post-injury were quite high. Highest observed mortalities and unfavourable outcomes were in the retired group, students had the best outcomes, followed by white-collar workers. Blue-collar, white-collar and unemployed patients showed similar patterns. Overall, 45% of patients died, and 55% had unfavourable outcome at 6 months post-injury.

**Table 4:** Outcomes of TBI patients by occupational status at different time points post-injury

Variable	Occupational status						Total (*n* = 886)	*P* value
	Blue-collar (*n* = 168)	White-collar (*n* = 105)	Entrepreneur (*n* = 93)	Retired (*n* = 213)	Unemployed (*n* = 134)	Student (*n* = 173)		
ICU mortality (*N*, %)	59 (35%)	31 (30%)	37 (40%)	123 (58%)	48 (36%)	51 (29%)	349 (39%)	< 0.001
Hospital mortality (*N*, %)	62 (37%)	32 (30%)	38 (41%)	139 (65%)	55 (41%)	54 (31%)	380 (43%)	< 0.001
6 months mortality (*N*, %)	62 (37%)	35 (33%)	38 (41%)	148 (69%)	58 (43%)	55 (32%)	396 (45%)	< 0.001
6 months unfavourable outcome (*N*, %)	80 (48%)	55 (52%)	44 (47%)	170 (80%)	73 (54%)	64 (37%)	486 (55%)	< 0.001

*ICU* intensive care unit

In order to analyse the association between occupational status and outcomes, multivariable regression models were constructed, using age, GCS and ISS as adjusting variables (Table 5). Defining the retired group as reference, all groups had higher odds of favourable outcome at 6 months post-injury—OR ranging from 2.2 (95% CI 1.1–4.6) for the entrepreneur group to 3.6 (95% CI 1.8–7.2) in the blue-collar group. Unemployed, blue-collar and white-collar workers had significantly higher odds of surviving at hospital discharge and at 6 months, and only unemployed and white-collar workers had significantly better odds of surviving at ICU discharge, compared to retired.

**Table 5:** Crude and adjusted odds ratios of the associations of occupational class with outcomes of TBI patients at different time points post-injury

Predictors	ICU outcome (alive = 1)		Hospital outcome (alive = 1)		6 Months outcome (alive = 1)		6 Months outcome (favourable = 1)	
	OR (95% CI)	*P* value	OR (95% CI)	*P* value	OR (95% CI)	*P *value	OR (95% CI)	*P * value
Occupational status								
Retired	1		1		1		1	
Unemployed	2.2 (1.2–4)	< 0.01	2.4 (1.3–4.3)	< 0.01	2.5 (1.4–4.6)	< 0.01	3.1 (1.7–5.9)	< 0.001
Entrepreneur	0.8 (0.4–1.6)	0.506	1.1 (0.6–2.3)	0.751	1.3 (0.7–2.7)	0.442	2.2 (1.1–4.6)	< 0.05
Blue-collar	1.7 (0.9–3.3)	0.107	2.3 (1.2–4.4)	< 0.05	2.6 (1.3–5.1)	< 0.01	3.6 (1.8–7.2)	< 0.001
White-collar	2.3 (1.1–4.8)	< 0.05	3.1 (1.5–6.5)	< 0.01	3 (1.4–6.2)	< 0.01	2.6 (1.2–5.5)	< 0.05
Student	1.4 (0.6–3.4)	0.494	1.7 (0.7–4.2)	0.239	1.7 (0.7–4.2)	0.238	3.5 (1.4–9.0)	< 0.01
Age	0.97 (0.95–0.98)	< 0.001	0.97 (0.95–0.98)	< 0.001	0.96 (0.95–0.98)	< 0.001	0.97 (0.95–0.98)	< 0.001
First GCS	1.5 (1.4–1.6)	< 0.001	1.5 (1.4–1.6)	< 0.001	1.5 (1.4–1.6)	< 0.001	1.5 (1.4–1.6)	< 0.001
ISS	0.95 (0.94–0.96)	< 0.001	0.95 (0.94–0.96)	< 0.001	0.95 (0.94–0.96)	< 0.001	0.95 (0.94–0.96)	< 0.001
Model parameters	Nagelkerke *R*^2^: 0.438 AUC: 0.842		Nagelkerke *R*^2^: 0.441 AUC: 0.841		Nagelkerke *R*^2^: 0.455 AUC: 0.847		Nagelkerke *R*^2^: 0.471 AUC: 0.853	

*ICU* intensive care unit, *GCS* Glasgow Coma Scale, *ISS* Injury Severity Score, *OR* odds ratio, *CI* confidence interval, *AUC* area under the curve

## Discussion

We conducted a study comparing demographic characteristics of patients, injury characteristics and outcomes of 886 patients with severe TBI after dividing them into six occupational groups. We found that patients displayed different patterns as to injury mechanism, severity and outcome among the compared groups. Occupational group predicted the outcome 6 months post-injury after adjusting for age and injury severity. Short-term outcomes (at ICU discharge and hospital discharge), and mortality at 6 months were less associated with occupational grouping.

To our knowledge, this is the first study analysing occupational status as a possible predictor of TBI patterns and outcomes to date. Therefore, direct comparison with published studies is not possible. Studies previously published on related topics mostly focused on socioeconomic status in general, measured in different ways and using various endpoints in the analyses. The study of Haines et al. [10] used ethnicity, race and insurance status and found that they are associated with differences in mortality, length of stay and discharge to inpatient rehabilitation. Hoofien et al. [11] in their study concluded that socioeconomic variables predicted various aspects of long-term functioning (14 years post-injury). Our study similarly showed varying mortality between the occupational groups as well as length of stay. Contrary to these studies, in a recent analysis by Zuckerman et al. [13], no associations were found between six SES variables and symptom duration or missed practice in a cohort of 282 student athletes with concussion.

In more general terms, better SES has been shown to be associated with improved non-fatal outcomes after injury in a recent review of literature conducted by Kruithof et al. [8], Loberg et al. [25] found higher mortality after trauma in patients living in high-poverty neighbourhoods, in African American patients, and in those enrolled in public health insurance. Gilbride et al. [26] found varying patterns of injury types in relation to SES, whereas Amram et al. [27] found that high rates of injuries were predicted by lower education levels of the studied area. McHale and colleagues [15] in their paper concluded that in less severe trauma, patients with low SES have increased risk of 30-day mortality. Thus, there is sufficient evidence supporting the general hypotheses that SES (measured in various ways) is an important factor to consider in the prevention of injuries and TBI and their treatment.

### Limitations of the study

We are aware of some limitations pertaining to this study. First, the data comes from different countries and different centres. This on one hand may bias our findings, as demographic characteristics of the populations of the included countries may differ, but it can also in a way be in favour of the generalizability of the findings—as they come from a more diverse population. Secondly, occupational status in our paper serves as a proxy measure of SES. We did not have the opportunity to assess the SES of patients in a more robust way due to non-availability of data. However, occupational characteristics are in general accepted as a good proxy for overall SES and have been used as such previously [7].

## Conclusion

Our paper provides clues pertaining specifically to variations in patterns and outcomes of TBI according to occupational status which can inform prevention and planning of services and can serve to plan priorities for further research.
